# Poly[[[μ_2_-1,1′-(butane-1,4-di­yl)bis­(1*H*-imidazole)-κ^2^ *N* ^3^:*N* ^3′^](μ_2_-2,6-di­methyl­pyridine-3,5-dicarboxyl­ato-κ^2^ *O* ^3^:*O* ^5^)zinc] dihydrate]

**DOI:** 10.1107/S1600536811039481

**Published:** 2011-09-30

**Authors:** Yu-Mei Yue, Lei Qian, Zheng-Hao Zhu, Cheng Wang, Ting Gao

**Affiliations:** a Pesticide Engineering Research Center of Heilongjiang Province, Heilongjiang University, Harbin 150080, People’s Republic of China; b Daqing Oil Field Water Supply Company, Institute of Source of Water Researching, Daqing 163458, People’s Republic of China; c Department of Service, Dionex China Ltd, Beijing 100029, People’s Republic of China; d School of Chemistry and Materials Science, Heilongjiang University, Harbin 150080, People’s Republic of China

## Abstract

In the title coordination polymer, {[Zn(C_9_H_7_NO_4_)(C_10_H_14_N_4_)]·2H_2_O}_*n*_, the Zn^II^ ion displays a distorted tetra­hedral geometry with two imidazole N atoms from two 1,1′-(butane-1,4-di­yl)bis­(imidazole) (bbi) ligands and two carboxyl­ate O atoms from two 2,6-dimethyl­pyridine-3,5-dicarboxyl­ate (dpdc) ligands. The bbi and dpdc ligands bridge the Zn^II^ ions, forming layers parallel to (011). O—H⋯O and O—H⋯N hydrogen bonds and π–π inter­actions between the imidazole rings [centroid–centroid distance = 3.807 (5) Å] connect the layers. Two of the three uncoordinated water mol­ecules are disordered, each over two 0.25-occupancy positions.

## Related literature

For transition metal complexes derived from 2,6-dimethyl­pyridine-3,5-dicarb­oxy­lic acid, see: Chen *et al.* (2009 ▶); Huang *et al.* (2008 ▶); Zhang *et al.* (2008*a* ▶); Zhou *et al.* (2009 ▶). For metal complexes derived from 1,1′-(butane-1,4-di­yl)bis­(imidazole) and carb­oxy­lic acids, see: Lan *et al.* (2008 ▶); Tian *et al.* (2009 ▶); Zhang *et al.* (2008*b* ▶).
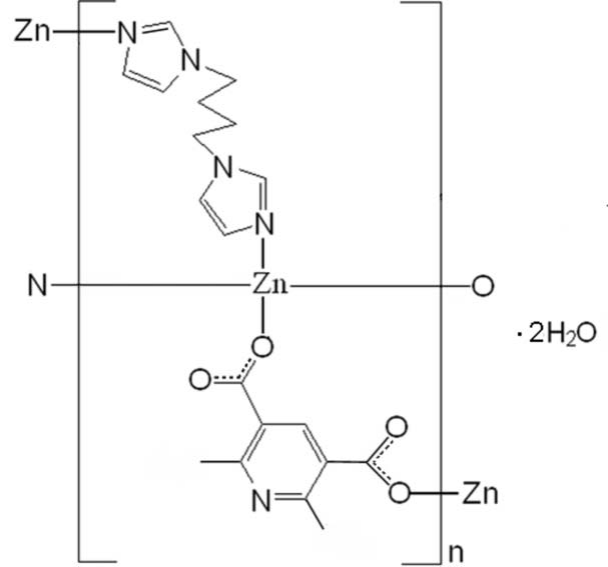


## Experimental

### 

#### Crystal data

[Zn(C_9_H_7_NO_4_)(C_10_H_14_N_4_)]·2H_2_O
*M* *_r_* = 484.82
Orthorhombic, 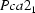

*a* = 17.8088 (12) Å
*b* = 9.4003 (4) Å
*c* = 15.5798 (8) Å
*V* = 2608.2 (2) Å^3^
*Z* = 4
Mo *K*α radiation
μ = 0.98 mm^−1^
*T* = 293 K
0.22 × 0.21 × 0.20 mm

#### Data collection

Bruker SMART APEX CCD diffractometer
Absorption correction: multi-scan (*SADABS*; Sheldrick, 1996 ▶) *T*_min_ = 0.807, *T*_max_ = 0.823
14228 measured reflections
4717 independent reflections
3753 reflections with *I* > 2σ(*I*)
*R*_int_ = 0.087

#### Refinement

*R*[*F* ^2^ > 2σ(*F* ^2^)] = 0.062
*wR*(*F* ^2^) = 0.175
*S* = 1.03
4717 reflections
315 parameters
36 restraints
H atoms treated by a mixture of independent and constrained refinement
Δρ_max_ = 0.67 e Å^−3^
Δρ_min_ = −0.64 e Å^−3^
Absolute structure: Flack (1983 ▶), 2186 Friedel pairs
Flack parameter: 0.03 (2)

### 

Data collection: *SMART* (Bruker, 2007 ▶); cell refinement: *SAINT* (Bruker, 2007 ▶); data reduction: *SAINT*; program(s) used to solve structure: *SHELXTL* (Sheldrick, 2008 ▶); program(s) used to refine structure: *SHELXTL*; molecular graphics: *XP* in *SHELXTL* and *DIAMOND* (Brandenburg, 1999 ▶); software used to prepare material for publication: *SHELXTL*.

## Supplementary Material

Crystal structure: contains datablock(s) I, global. DOI: 10.1107/S1600536811039481/hy2452sup1.cif

Structure factors: contains datablock(s) I. DOI: 10.1107/S1600536811039481/hy2452Isup2.hkl

Additional supplementary materials:  crystallographic information; 3D view; checkCIF report

## Figures and Tables

**Table 1:** Selected bond lengths (Å)

Zn1—O1	1.954 (4)
Zn1—O4^i^	1.965 (3)
Zn1—N2	1.994 (6)
Zn1—N4^ii^	2.032 (6)

Symmetry codes: (i) 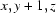; (ii) 
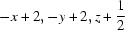
.

**Table 2:** Hydrogen-bond geometry (Å, °)

*D*—H⋯*A*	*D*—H	H⋯*A*	*D*⋯*A*	*D*—H⋯*A*
O1*W*—H1*B*⋯O2^iii^	0.87 (4)	1.99 (4)	2.820 (9)	160 (4)
O1*W*—H1*A*⋯N1	0.87 (3)	1.95 (3)	2.736 (10)	149 (5)

Symmetry code: (iii) 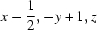.

**Fig. 1.**
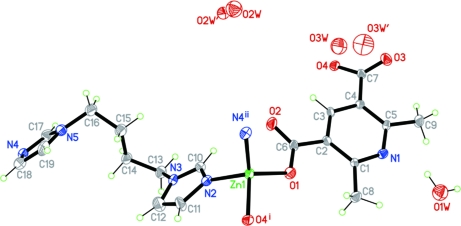
The asymmetric unit of the title compound. Displacement ellipsoids are drawn at the 30% probability level. [Symmetry codes: (i) x, 1+y, z; (ii) 2-x, 2-y, 0.5+z.]

**Fig. 2.**
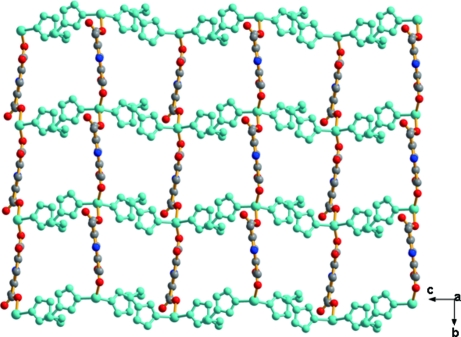
The crystal packing of the title compound, showing the layer structure.

## supplementary crystallographic information

### Comment

In recent years, great interest has been focused on the crystal engineering of coordination frameworks. As is already known, pyridine-3,5-dicarboxylic acid is a rigid and linear ligand that possesses the capability to bridge metal atoms in various coordination modes through the carboxylate O atoms and the pyridine N atom (Chen *et al.*, 2009; Huang *et al.*, 2008; Zhang *et al.*, 2008*a*; Zhou *et al.*, 2009). Flexible 1,1'-(butane-1,4-diyl)bis(imidazole) (bbi) ligand with organic carboxylic acids can build diverse topological architectures (Lan *et al.*, 2008; Tian *et al.*, 2009; Zhang *et al.*, 2008*b*). Herein, we report the crystal structure of the title compound obtained by reacting bbi and 2,6-dimethylpyridine-3,5-dicarboxylic acid (H_2_dpdc) with Zn(NO_3_)_2_.

In the title compound (Fig. 1), the Zn^II^ ion has a distorted tetrahedral geometry with two imidazole N atoms from two different bbi ligands and two carboxylate O atoms from two different dpdc ligands (Table 1). The bbi and dpdc ligands bridge the Zn^II^ ions into a layer parallel to (0 1 1) (Fig. 2). O—H···O and O—H···N hydrogen bonds (Table 2) and π–π interactions between the imidazole rings [centroid–centroid distance = 3.807 (5) Å] connect the layers.

### Experimental

The title complex was obtained by the reaction of zinc(II) nitrate (59.5 mg, 0.2 mmol) with bbi (37.6 mg, 0.2 mmol) and H_2_dpdc (39.4 mg, 0.2 mmol) in DMF/ethanol/water (10/10/5 ml). The mixture was stirred for 1 h and the solution was placed at room temperature for solvent volatilization. Single crystals were obtained after several days. Analysis, calculated for C_19_H_25_N_5_O_6_Zn: C 47.96, H 5.08, N 14.72%; found: C 48.08, H 5.49, N 14.52%.

### Refinement

H atoms attached to C atoms were positioned geometrically and refined as riding atoms, with C—H = 0.93 (aromatic), 0.97 (methylene) and 0.96 (methyl) Å and with *U*_iso_(H) = 0.08 Å^2^. H atoms on O1W were located in a difference Fourier map and refined with a distance restraint of O—H = 0.85 Å and *U*_iso_(H) = 0.08 Å^2^. H atom on disordered O2W, O2W', O3W and O3W' were not located duo to partial possession of the H atoms.

### Crystal data

**Table:**

[Zn(C_9_H_7_NO_4_)(C_10_H_14_N_4_)]·2H_2_O	*D*_x_ = 1.234 Mg m^−^^3^
*M**_r_* = 484.82	Melting point: not measured K
Orthorhombic, *P**c**a*2_1_	Mo *K*α radiation, λ = 0.71073 Å
Hall symbol: P 2c -2ac	Cell parameters from 4717 reflections
*a* = 17.8088 (12) Å	θ = 2.3–25.5°
*b* = 9.4003 (4) Å	µ = 0.98 mm^−^^1^
*c* = 15.5798 (8) Å	*T* = 293 K
*V* = 2608.2 (2) Å^3^	Block, colorless
*Z* = 4	0.22 × 0.21 × 0.20 mm
*F*(000) = 1008	

### Data collection

**Table:**

Bruker SMART APEX CCD diffractometer	4717 independent reflections
Radiation source: fine-focus sealed tube	3753 reflections with *I* > 2σ(*I*)
graphite	*R*_int_ = 0.087
φ and ω scans	θ_max_ = 25.5°, θ_min_ = 2.3°
Absorption correction: multi-scan (*SADABS*; Sheldrick, 1996)	*h* = −12→21
*T*_min_ = 0.807, *T*_max_ = 0.823	*k* = −11→11
14228 measured reflections	*l* = −18→18

### Refinement

**Table:**

Refinement on *F*^2^	Secondary atom site location: difference Fourier map
Least-squares matrix: full	Hydrogen site location: inferred from neighbouring sites
*R*[*F*^2^ > 2σ(*F*^2^)] = 0.062	H atoms treated by a mixture of independent and constrained refinement
*wR*(*F*^2^) = 0.175	*w* = 1/[σ^2^(*F*_o_^2^) + (0.1192*P*)^2^] where *P* = (*F*_o_^2^ + 2*F*_c_^2^)/3
*S* = 1.03	(Δ/σ)_max_ = 0.042
4717 reflections	Δρ_max_ = 0.67 e Å^−^^3^
315 parameters	Δρ_min_ = −0.64 e Å^−^^3^
36 restraints	Absolute structure: Flack (1983), 2186 Friedel pairs
Primary atom site location: structure-invariant direct methods	Flack parameter: 0.03 (2)

### Fractional atomic coordinates and isotropic or equivalent isotropic displacement parameters (Å^2^)

**Table:**

	*x*	*y*	*z*	*U*_iso_*/*U*_eq_	Occ. (<1)
Zn1	0.73885 (3)	0.97189 (5)	0.87767 (6)	0.0381 (2)	
O1	0.6630 (2)	0.8258 (4)	0.8567 (3)	0.0560 (12)	
O2	0.7521 (2)	0.6656 (5)	0.8691 (6)	0.0684 (15)	
O3	0.5991 (3)	0.1007 (5)	0.9535 (4)	0.0695 (14)	
O4	0.6934 (2)	0.1619 (3)	0.8694 (3)	0.0467 (9)	
C3	0.6499 (2)	0.4454 (4)	0.8818 (5)	0.0351 (10)	
H3	0.7012	0.4269	0.8817	0.080*	
N2	0.8088 (3)	0.9860 (6)	0.7781 (4)	0.0429 (13)	
N3	0.8753 (3)	0.9276 (6)	0.6618 (4)	0.0522 (13)	
N4	1.2098 (4)	1.0326 (6)	0.4941 (4)	0.0477 (15)	
N5	1.1556 (4)	0.9499 (7)	0.6067 (3)	0.0527 (16)	
C2	0.6262 (3)	0.5836 (5)	0.8704 (4)	0.0362 (10)	
C1	0.5488 (2)	0.6115 (5)	0.8722 (4)	0.0362 (10)	
N1	0.5019 (2)	0.4987 (4)	0.8838 (6)	0.0395 (10)	
C5	0.5237 (3)	0.3608 (6)	0.8968 (3)	0.0381 (13)	
C4	0.5999 (3)	0.3304 (5)	0.8935 (3)	0.0363 (13)	
C6	0.6854 (3)	0.6980 (5)	0.8651 (4)	0.0383 (12)	
C7	0.6308 (3)	0.1830 (5)	0.9067 (3)	0.0372 (12)	
C8	0.5117 (3)	0.7510 (6)	0.8585 (5)	0.0523 (16)	
H8A	0.5160	0.7777	0.7992	0.080*	
H8B	0.5354	0.8218	0.8937	0.080*	
H8C	0.4596	0.7439	0.8738	0.080*	
C9	0.4619 (4)	0.2569 (7)	0.9113 (5)	0.0599 (18)	
H9A	0.4456	0.2623	0.9700	0.080*	
H9B	0.4796	0.1625	0.8993	0.080*	
H9C	0.4206	0.2789	0.8740	0.080*	
C10	0.8374 (4)	0.8823 (7)	0.7308 (4)	0.0470 (15)	
H10	0.8318	0.7865	0.7444	0.080*	
C11	0.8304 (5)	1.1069 (9)	0.7376 (5)	0.065 (2)	
H11	0.8187	1.1980	0.7567	0.080*	
C12	0.8718 (5)	1.0785 (10)	0.6648 (6)	0.073 (2)	
H12	0.8927	1.1432	0.6264	0.080*	
C13	0.9116 (4)	0.8468 (8)	0.5972 (4)	0.0553 (16)	
H13A	0.8888	0.8685	0.5422	0.080*	
H13B	0.9035	0.7465	0.6086	0.080*	
C14	0.9961 (4)	0.8745 (8)	0.5913 (4)	0.0560 (16)	
H14A	1.0183	0.8045	0.5532	0.080*	
H14B	1.0041	0.9675	0.5659	0.080*	
C15	1.0364 (4)	0.8687 (9)	0.6769 (4)	0.0663 (19)	
H15A	1.0157	0.7917	0.7109	0.080*	
H15B	1.0277	0.9569	0.7076	0.080*	
C16	1.1211 (4)	0.8462 (11)	0.6664 (5)	0.080 (3)	
H16A	1.1451	0.8545	0.7221	0.080*	
H16B	1.1301	0.7507	0.6452	0.080*	
C17	1.1862 (5)	0.9177 (9)	0.5306 (5)	0.060 (2)	
H17	1.1900	0.8266	0.5077	0.080*	
C18	1.1924 (4)	1.1407 (8)	0.5463 (4)	0.0531 (16)	
H18	1.2017	1.2365	0.5358	0.080*	
C19	1.1601 (4)	1.0882 (12)	0.6143 (5)	0.069 (2)	
H19	1.1429	1.1408	0.6609	0.080*	
O1W	0.3529 (5)	0.5563 (7)	0.9086 (4)	0.0930 (19)	
O3W	0.7170 (19)	0.455 (3)	1.0985 (19)	0.094 (7)	0.25
O3W'	0.670 (3)	0.304 (5)	1.064 (3)	0.166 (14)	0.25
O2W'	0.9930 (11)	0.4765 (19)	1.0714 (12)	0.053 (4)	0.25
O2W	0.9724 (18)	0.538 (3)	1.1357 (19)	0.091 (7)	0.25
H1B	0.320 (2)	0.488 (4)	0.910 (3)	0.080*	
H1A	0.3963 (11)	0.515 (6)	0.916 (4)	0.080*	

### Atomic displacement parameters (Å^2^)

**Table:**

	*U*^11^	*U*^22^	*U*^33^	*U*^12^	*U*^13^	*U*^23^
Zn1	0.0316 (3)	0.0328 (3)	0.0500 (3)	−0.0002 (2)	0.0009 (4)	0.0007 (5)
O1	0.044 (2)	0.0301 (18)	0.094 (4)	−0.0058 (15)	−0.003 (2)	0.003 (2)
O2	0.037 (2)	0.042 (2)	0.126 (5)	−0.0053 (15)	0.003 (3)	0.012 (4)
O3	0.070 (3)	0.048 (3)	0.091 (3)	0.011 (2)	0.024 (3)	0.023 (3)
O4	0.0391 (19)	0.0318 (16)	0.069 (2)	0.0063 (13)	0.002 (2)	0.005 (2)
C3	0.032 (2)	0.033 (2)	0.040 (2)	−0.0028 (17)	0.009 (3)	0.005 (3)
N2	0.034 (3)	0.045 (3)	0.050 (3)	0.003 (2)	−0.002 (2)	0.001 (2)
N3	0.029 (3)	0.062 (3)	0.066 (3)	0.002 (3)	0.004 (2)	0.012 (3)
N4	0.037 (3)	0.054 (3)	0.052 (3)	0.000 (2)	0.001 (2)	0.002 (3)
N5	0.046 (3)	0.072 (4)	0.041 (3)	0.002 (3)	0.002 (2)	0.008 (3)
C2	0.036 (2)	0.034 (2)	0.039 (3)	−0.0024 (19)	0.005 (3)	0.004 (3)
C1	0.031 (2)	0.037 (2)	0.041 (2)	0.0024 (18)	0.006 (3)	0.009 (3)
N1	0.035 (2)	0.036 (2)	0.047 (3)	−0.0004 (15)	−0.007 (3)	−0.004 (2)
C5	0.028 (2)	0.042 (3)	0.045 (4)	−0.008 (2)	−0.001 (2)	0.002 (2)
C4	0.035 (3)	0.028 (2)	0.046 (4)	0.0025 (19)	0.005 (2)	0.003 (2)
C6	0.038 (3)	0.030 (2)	0.048 (3)	−0.0060 (19)	0.004 (2)	−0.001 (2)
C7	0.040 (3)	0.030 (3)	0.042 (3)	−0.002 (2)	0.004 (2)	0.004 (2)
C8	0.039 (3)	0.038 (3)	0.080 (5)	0.003 (2)	−0.002 (3)	0.001 (3)
C9	0.040 (3)	0.047 (3)	0.092 (5)	−0.014 (3)	0.002 (3)	0.008 (3)
C10	0.040 (4)	0.046 (3)	0.055 (3)	−0.001 (3)	−0.004 (3)	0.008 (3)
C11	0.068 (5)	0.050 (4)	0.076 (5)	0.001 (4)	0.035 (4)	0.012 (4)
C12	0.076 (6)	0.063 (5)	0.081 (5)	−0.005 (5)	0.015 (4)	0.002 (4)
C13	0.048 (4)	0.071 (4)	0.047 (3)	0.000 (3)	0.007 (3)	−0.002 (3)
C14	0.040 (3)	0.078 (5)	0.050 (3)	0.004 (3)	0.009 (3)	0.010 (3)
C15	0.051 (4)	0.099 (6)	0.049 (4)	−0.002 (4)	0.004 (3)	0.014 (4)
C16	0.048 (4)	0.125 (8)	0.066 (4)	0.001 (4)	0.005 (3)	0.038 (5)
C17	0.056 (4)	0.062 (5)	0.063 (4)	0.022 (4)	0.006 (3)	0.007 (4)
C18	0.048 (4)	0.059 (4)	0.052 (3)	0.009 (3)	0.005 (3)	−0.013 (3)
C19	0.041 (4)	0.102 (7)	0.063 (5)	−0.002 (5)	0.008 (3)	−0.012 (4)
O1W	0.090 (2)	0.092 (2)	0.097 (2)	−0.0007 (10)	0.0000 (10)	−0.0017 (10)
O3W	0.094 (7)	0.094 (7)	0.093 (7)	0.0002 (10)	−0.0002 (10)	0.0003 (10)
O3W'	0.166 (14)	0.166 (14)	0.166 (14)	0.0000 (10)	0.0000 (10)	0.0001 (10)
O2W'	0.053 (4)	0.053 (4)	0.053 (4)	0.0006 (10)	0.0003 (10)	−0.0006 (10)
O2W	0.092 (7)	0.091 (7)	0.092 (7)	0.0001 (10)	0.0002 (10)	0.0001 (10)

### Geometric parameters (Å, °)

**Table:**

Zn1—O1	1.954 (4)	C4—C7	1.505 (7)
Zn1—O4^i^	1.965 (3)	C8—H8A	0.9600
Zn1—N2	1.994 (6)	C8—H8B	0.9600
Zn1—N4^ii^	2.032 (6)	C8—H8C	0.9600
O1—C6	1.272 (6)	C9—H9A	0.9600
O2—C6	1.228 (7)	C9—H9B	0.9600
O3—C7	1.204 (7)	C9—H9C	0.9600
O4—C7	1.273 (7)	C10—H10	0.9300
O4—Zn1^iii^	1.965 (3)	C11—C12	1.380 (12)
C3—C2	1.377 (7)	C11—H11	0.9300
C3—C4	1.412 (6)	C12—H12	0.9300
C3—H3	0.9300	C13—C14	1.530 (9)
N2—C10	1.324 (9)	C13—H13A	0.9700
N2—C11	1.355 (10)	C13—H13B	0.9700
N3—C10	1.339 (8)	C14—C15	1.515 (9)
N3—C13	1.418 (9)	C14—H14A	0.9700
N3—C12	1.420 (11)	C14—H14B	0.9700
N4—C17	1.291 (11)	C15—C16	1.533 (11)
N4—C18	1.337 (9)	C15—H15A	0.9700
N4—Zn1^iv^	2.032 (6)	C15—H15B	0.9700
N5—C19	1.308 (12)	C16—H16A	0.9700
N5—C17	1.340 (9)	C16—H16B	0.9700
N5—C16	1.480 (9)	C17—H17	0.9300
C2—C1	1.403 (6)	C18—C19	1.303 (10)
C2—C6	1.509 (7)	C18—H18	0.9300
C1—N1	1.362 (6)	C19—H19	0.9300
C1—C8	1.484 (7)	O1W—H1B	0.870 (10)
N1—C5	1.368 (7)	O1W—H1A	0.871 (10)
C5—C4	1.388 (7)	O3W—O3W'	1.73 (6)
C5—C9	1.489 (8)	O2W'—O2W	1.21 (3)
			
O1—Zn1—O4^i^	110.09 (16)	C5—C9—H9B	109.5
O1—Zn1—N2	110.4 (2)	H9A—C9—H9B	109.5
O4^i^—Zn1—N2	98.4 (2)	C5—C9—H9C	109.5
O1—Zn1—N4^ii^	116.5 (2)	H9A—C9—H9C	109.5
O4^i^—Zn1—N4^ii^	105.2 (2)	H9B—C9—H9C	109.5
N2—Zn1—N4^ii^	114.5 (3)	N2—C10—N3	114.0 (6)
C6—O1—Zn1	115.4 (4)	N2—C10—H10	123.0
C7—O4—Zn1^iii^	118.2 (3)	N3—C10—H10	123.0
C2—C3—C4	123.0 (4)	N2—C11—C12	111.8 (7)
C2—C3—H3	118.5	N2—C11—H11	124.1
C4—C3—H3	118.5	C12—C11—H11	124.1
C10—N2—C11	104.4 (6)	C11—C12—N3	104.1 (7)
C10—N2—Zn1	128.6 (5)	C11—C12—H12	128.0
C11—N2—Zn1	126.5 (5)	N3—C12—H12	128.0
C10—N3—C13	129.0 (6)	N3—C13—C14	113.7 (6)
C10—N3—C12	105.7 (5)	N3—C13—H13A	108.8
C13—N3—C12	125.3 (6)	C14—C13—H13A	108.8
C17—N4—C18	107.0 (7)	N3—C13—H13B	108.8
C17—N4—Zn1^iv^	121.5 (5)	C14—C13—H13B	108.8
C18—N4—Zn1^iv^	131.5 (5)	H13A—C13—H13B	107.7
C19—N5—C17	106.3 (6)	C15—C14—C13	114.0 (5)
C19—N5—C16	128.5 (6)	C15—C14—H14A	108.8
C17—N5—C16	125.2 (7)	C13—C14—H14A	108.8
C3—C2—C1	118.4 (4)	C15—C14—H14B	108.8
C3—C2—C6	117.7 (4)	C13—C14—H14B	108.8
C1—C2—C6	123.6 (4)	H14A—C14—H14B	107.7
N1—C1—C2	117.3 (4)	C14—C15—C16	112.2 (5)
N1—C1—C8	115.7 (4)	C14—C15—H15A	109.2
C2—C1—C8	126.9 (4)	C16—C15—H15A	109.2
C1—N1—C5	125.7 (4)	C14—C15—H15B	109.2
N1—C5—C4	117.8 (4)	C16—C15—H15B	109.2
N1—C5—C9	115.8 (5)	H15A—C15—H15B	107.9
C4—C5—C9	126.4 (5)	N5—C16—C15	112.6 (6)
C5—C4—C3	117.6 (4)	N5—C16—H16A	109.1
C5—C4—C7	122.8 (5)	C15—C16—H16A	109.1
C3—C4—C7	119.4 (4)	N5—C16—H16B	109.1
O2—C6—O1	122.9 (5)	C15—C16—H16B	109.1
O2—C6—C2	119.7 (4)	H16A—C16—H16B	107.8
O1—C6—C2	117.4 (5)	N4—C17—N5	109.5 (7)
O3—C7—O4	126.0 (5)	N4—C17—H17	125.3
O3—C7—C4	120.2 (5)	N5—C17—H17	125.3
O4—C7—C4	113.7 (4)	C19—C18—N4	108.0 (8)
C1—C8—H8A	109.5	C19—C18—H18	126.0
C1—C8—H8B	109.5	N4—C18—H18	126.0
H8A—C8—H8B	109.5	C18—C19—N5	109.2 (7)
C1—C8—H8C	109.5	C18—C19—H19	125.4
H8A—C8—H8C	109.5	N5—C19—H19	125.4
H8B—C8—H8C	109.5	H1B—O1W—H1A	105 (4)
C5—C9—H9A	109.5		

Symmetry codes: (i) *x*, *y*+1, *z*; (ii) −*x*+2, −*y*+2, *z*+1/2; (iii) *x*, *y*−1, *z*; (iv) −*x*+2, −*y*+2, *z*−1/2.

### Hydrogen-bond geometry (Å, °)

**Table:**

*D*—H···*A*	*D*—H	H···*A*	*D*···*A*	*D*—H···*A*
O1W—H1B···O2^v^	0.87 (4)	1.99 (4)	2.820 (9)	160 (4)
O1W—H1A···N1	0.87 (3)	1.95 (3)	2.736 (10)	149 (5)

Symmetry codes: (v) *x*−1/2, −*y*+1, *z*.
